# Climate change will affect global water availability through compounding changes in seasonal precipitation and evaporation

**DOI:** 10.1038/s41467-020-16757-w

**Published:** 2020-06-23

**Authors:** Goutam Konapala, Ashok K. Mishra, Yoshihide Wada, Michael E. Mann

**Affiliations:** 1 Glenn Department of Civil Engineering, Lowry Hall, Clemson University, Clemson, SC 29634-0911 USA; 2 Environmental Sciences Division, Oak Ridge National Laboratory, Oak Ridge, TN 37831 USA; 3 Climate Change Science Institute, Oak Ridge National Laboratory, Oak Ridge, TN 37831 USA; 4 Water program, International Institute for Applied Systems Analysis, Schlossplatz 1, A-2361 Laxenburg, Austria; 5 Department of Meteorology and Atmospheric Science, 502 Walker Building, Pennsylvania State University, University Park, PA 16802 USA

**Keywords:** Climate sciences, Climate-change impacts, Projection and prediction, Hydrology, Water resources

## Abstract

Both seasonal and annual mean precipitation and evaporation influence patterns of water availability impacting society and ecosystems. Existing global climate studies rarely consider such patterns from non-parametric statistical standpoint. Here, we employ a non-parametric analysis framework to analyze seasonal hydroclimatic regimes by classifying global land regions into nine regimes using late 20th century precipitation means and seasonality. These regimes are used to assess implications for water availability due to concomitant changes in mean and seasonal precipitation and evaporation changes using CMIP5 model future climate projections. Out of 9 regimes, 4 show increased precipitation variation, while 5 show decreased evaporation variation coupled with increasing mean precipitation and evaporation. Increases in projected seasonal precipitation variation in already highly variable precipitation regimes gives rise to a pattern of “seasonally variable regimes becoming more variable”. Regimes with low seasonality in precipitation, instead, experience increased wet season precipitation.

## Abstract (web summary)

Adequate water availability is key to human and ecosystem sustainability. Here, the authors show that seasonally variable regimes become more variable, and the combined influence of seasonality and magnitude of climate variables will affect future water availability.

## Introduction

Accessibility of water resources for human consumption and ecosystems largely depends on the spatio-temporal distribution of both precipitation and evaporation^1–3^. As a result, changes in characteristics of precipitation and evaporation due to human-caused climate change in the 21st century may result in changes in water availability (WA) that have implications for both humans and the biosphere^4,5^. Previous studies have elucidated trends in precipitation in terms of both annual mean^6,7^, seasonal variation^8,9^, and the distribution of extreme events^10,11^. Studies have also examined the corresponding changes in evaporation characteristics^12–14^. Though the combined monthly distribution of precipitation and evaporation have widespread implications for regional hydrology^15,16^, crop yield^17,18^, and ecology^19,20^, few studies have examined the concomitant changes in both annual mean and seasonal variation in these variables. Moreover, the existing global climate classifications^21–23^ that form the basis for WA studies rarely consider seasonal variation characteristics from a non-parametric standpoint, even though they vary in a complex manner across global land regions^24,25^.

An analysis of the collective changes in both hydrological annual means and seasonal variations can better inform assessments of societal and ecological vulnerability with respect to potential future WA. For instance, an increase in seasonal variability of precipitation might possibly disrupt the continuous atmospheric water supply, leading to extended dry periods in regions of unimodal precipitation distribution^26,27^. In regimes with high precipitation, this redistribution may result in more water concentrated over relatively short periods of time, leading to floods and operational difficulties in reservoir water management. Similarly, an increase in seasonal variation in evaporation might lead to changes in the monthly terrestrial water budget depending on the water supply regime^28,29^. It is thus important to understand the combined role of projected future changes in both mean precipitation and evaporation and their seasonal variations in assessing impacts on any particular region’s water supply regime.

Here, we examine spatially aggregated future projections for nine distinct regimes, characterizing joint changes in annual mean and seasonal precipitation and evaporation. Each regime displays concomitant increases in annual mean precipitation and evaporation. However, only four of the nine regimes display increased seasonal variation in precipitation. We observe a tendency for increased seasonal variation for regimes that already exhibit high seasonal variability, establishing a pattern wherein seasonally variable regimes become more variable in the future. Regimes with low seasonality in precipitation, instead, experience increased wet season precipitation.

## Results

### Global classification of precipitation regimes

In this study, we first classify the global land regions into distinct hydroclimatic regimes based on annual means and seasonal variations using observed monthly gridded precipitation data from the Global Precipitation Climatology Centre (GPCC) (See “Methods”)^30^. For quantifying seasonality, we used apportionment entropy (AE), which provides a descriptive non-parametric measure determining the seasonal variation for data, such as precipitation, that are not Gaussian distributed, as it captures higher-order statistics unlike parametric methods that characterize the data in terms of, e.g., coefficient of variation and standard deviation^3^. In our case, higher AE values imply lower seasonal variation, and lower AE imply higher seasonal variation (see “Methods” for further details).

**Fig. 1:**
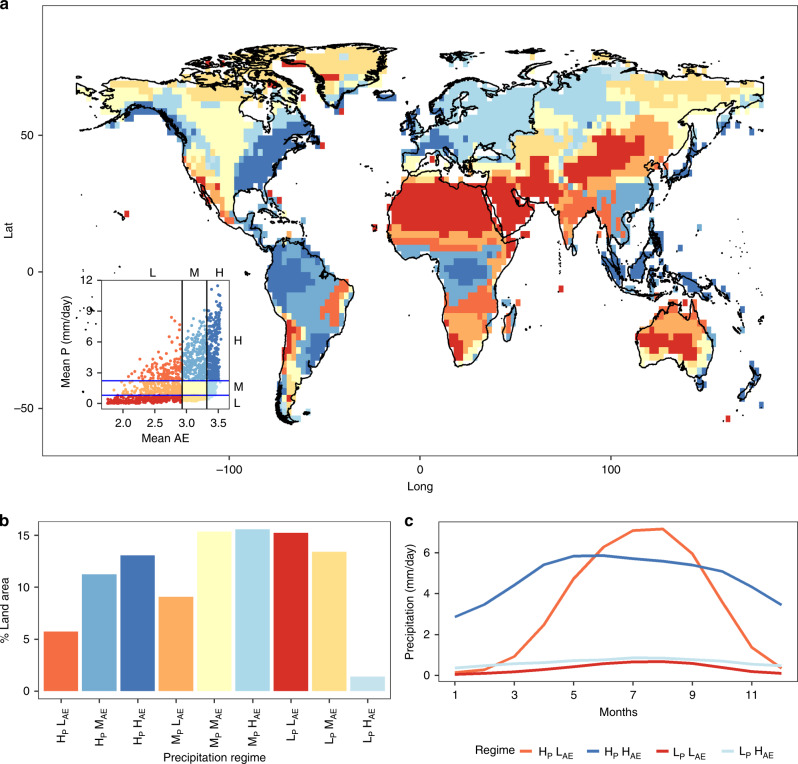
Classification of the precipitation regimes. **a** Spatial distribution of precipitation regimes based on the percentile (Pr_i_) thresholds concept using mean apportionment entropy (AE) and annual precipitation P during the 1971–2000 reference period. **b** Percentage of land occupied by each regime. **c** Precipitation climatology of the spatially aggregated monthly rainfall climatology for regimes H_P_L_AE_, H_P_H_AE_, L_P_H_AE_, L_P_L_AE_. These regimes are selected as they represent boundary case scenarios. H, M, and L represent high moderate and low values, respectively.

To understand the regime classification framework qualitatively, we plotted various characteristics of the resulting regimes in Fig. 1. The spatial distribution of regimes based on precipitation means and seasonal variations is shown in Fig. 1a. The percentage of land occupied by each classified regime is shown in Fig. 1b. The plot region in the legend is divided into nine zones, each of which is delineated with two intersecting dividing lines that pass through the limits of the respective thresholds of the two variables. Global land regions are classified into nine regimes based on percentiles thresholds (i.e., <30th—Low; 30–70th—Moderate; >70th—High) of seasonal variation (as defined by AE) and annual mean precipitation during the 1971–2000 reference period (See “Methods”). We chose four critical zones (H_p_H_AE_, L_P_H_AE_, L_P_L_AE_, H_P_L_AE_) to illustrate extreme scenarios based on combinations of either low (<Pr_30_) and high (>Pr_70_) variation and mean value of precipitation. Here, H_AE_ represents higher AE values, which implies lower seasonal variation. The spatially aggregate precipitation climatology of the selected four regimes is detailed in Fig. 1c. For each grid, the month with lowest rainfall is plotted as starting month for rainfall. Regime H_p_H_AE_ witnesses a higher precipitation rate and lower intra-annual variability (i.e., high AE). As a result, the precipitation is uniformly distributed, indicating perennial water supply for both human and ecological needs^3,31^. On the contrary, regime H_P_L_AE_ presents a scenario (high precipitation and high variability), where most of the precipitation is concentrated in a limited number of months. As a result, excess water needs to be stored to prevent floods as well as to enhance water supply for stakeholders in dry months. In low-precipitation regimes (L_P_H_AE_ and L_P_L_AE_), virtual water transfers and drought-tolerant crops are prevalent^32^. However, regions with less precipitation and higher AE (L_P_H_AE_) are perennial in nature. Regions characterized by a combination of lower precipitation and AE (L_P_L_AE_) are arid in nature, indicating water resources availability is extremely low. Therefore, based on these principles, the remaining regimes have precipitation conditions between these extreme limiting cases.

The spatial distribution of regimes across global land is found to be as follows: regime H_P_L_AE_ and M_P_L_AE_ can be found mostly in the Indian subcontinent, Northeast Asia, Northern Australia, and much of north-central and south-central Africa, covering land area of ~15%. These regions are influenced mostly by monsoons^33^. Regime H_P_M_AE_ and H_P_H_AE_ mostly occupy eastern North America, Northern South America, central Africa, Western Europe, and South Eastern Asia, occupying a combined land area of 24%. These regions are mostly moist forest areas. Regime M_P_M_AE_ spans across most of central North America and is scattered in western parts of South America and Northern and central Asia, Southern part of Africa and Southern Australia amounting to ~15% of land area. Regime M_P_H_AE_ occupies most of European Continent and further extends to western Russia occupying 16% of the total land area. Regime L_P_L_AE_ occupies Northern Africa, the Middle East, and further extends to central Asia. In addition, central Australia, the interior western United States and central western South America are classified as belonging to L_P_L_AE,_ occupying a land area of 15%. L_P_M_AE_ is mostly confined to the Northern parts of North America and Siberia region occupying ~13% of land area. Finally, regime L_P_H_AE_ is confined to a small region in the central Russian region.

### Trends in annual changes of precipitation and evaporation

Precipitation and evaporation projections from Coupled Model Intercomparison Project Phase 5 (CMIP5) models are aggregated over these nine precipitation regimes. We evaluate linear trends over the 21st century (2005–2100) using Bayesian model averaging (BMA) (31*–*32, see “Methods”) applied to both annual means and seasonal variation in precipitation and evaporation for three future scenarios (representative concentration pathway (RCP) 2.6, 4.5, and 8.5).

**Fig. 2:**
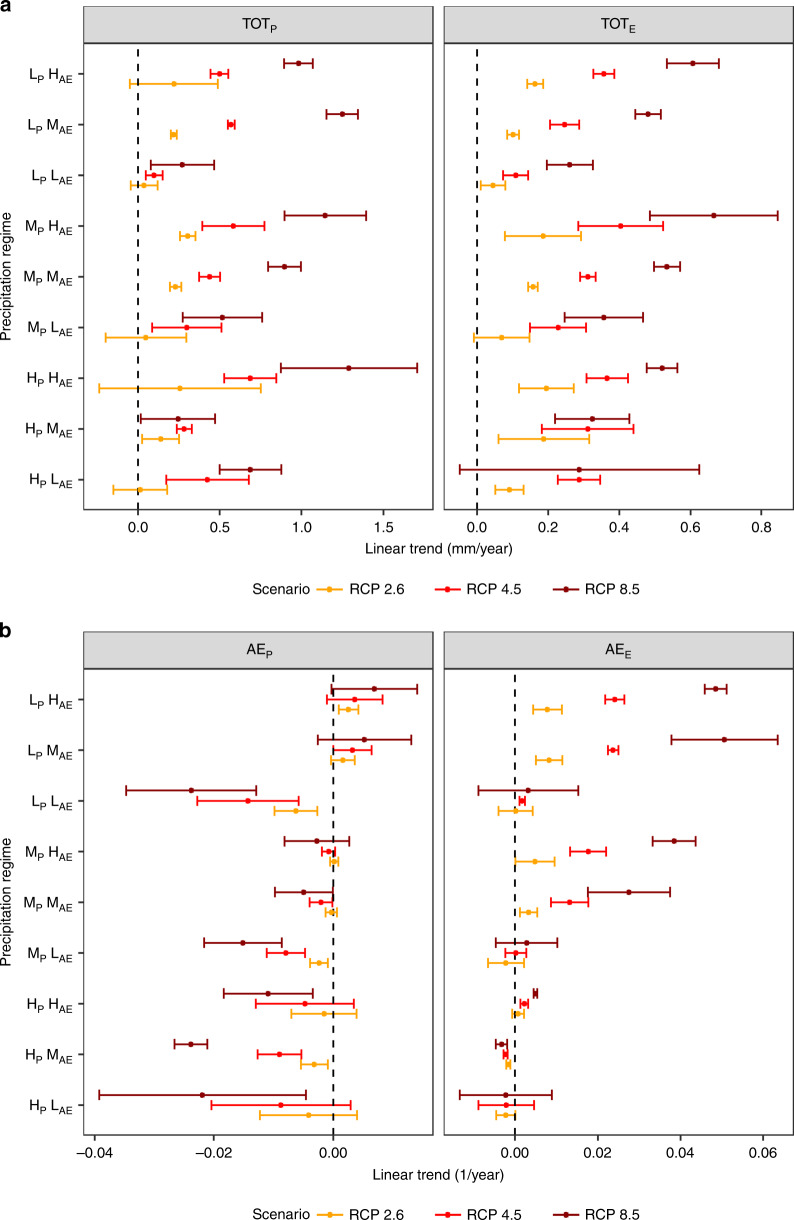
Trends in precipitation and evaporation totals and seasonality. Theil-sen slopes of **a** total annual precipitation (TOT_P_) and total annual evaporation (TOT_E_) magnitudes, and **b** precipitation apportionment entropy (AE_P_) and evaporation apportionment entropy AE_E_. The error bars surrounding the point estimates represent the 95% confidence interval of the CMIP5 multimodel ensemble. H, M, and L represent high moderate and low values, respectively.

The changes in BMA-weighted future annual precipitation and evaporation totals and seasonal variability for the RCP scenarios 2.6, 4.5, and 8.6 are shown in Fig. 2a, b, respectively (note that the horizontal axis scales are different for both panels in Fig. 2a, b). In Fig. 2a, the change in annual precipitation total (TOT_P_) indicates an increase in precipitation in all regimes, with the least increase in regime L_P_L_AE_, In addition, a proportional relationship was observed in terms of an increase in the precipitation magnitude with an increase in the emission forcing. In addition, in precipitation regimes L_P_H_AE_, M_P_H_AE_, and H_P_H_AE_ that are characterized by a relatively consistent water supply, the TOT_P_ exhibit a higher magnitude of precipitation increase compared with those regimes characterized by a moderate and high seasonal variability. Conversely, regions L_P_L_AE_, M_P_L_AE_, and H_P_L_AE_ with inconsistent water supplies exhibit a lower magnitude of precipitation increase. The highest magnitude increase is evident in regime H_P_H_AE_, which is characterized by high volumes of consistent water supply in which RCP 8.5 exhibits an increase of 1.3 mm/year, followed by RCP 4.5 exhibiting 0.7 mm/year and RCP 2.6 exhibiting 0.25 mm/year. In addition, a larger uncertainty in the case of GCM model use is evident in precipitation regime H_P_H_AE_. Similar to TOT_P_, an increase in the annual evaporation total (TOT_E_) is observed in all regimes with the lowest increase observed in regime L_P_L_AE_. In addition, a direct proportional relationship is also evident here; the higher emission scenarios exhibit a higher magnitude of increased evaporation. As in the case of TOT_P_, TOT_E_ in the precipitation regimes of L_P_H_AE_, M_P_H_AE_, and H_P_H_AE_ that are characterized by relatively consistent water supply exhibit a higher magnitude of evaporation increase. The greatest change is evident in regime M_P_H_AE_, which is characterized by moderate precipitation but low seasonal variability. In this regime, RCP 8.5 exhibits an increase of 0.68 mm/year, followed by RCP 4.5 with an increase of 0.4 mm/year and RCP 2.6 with an increase of 0.19 mm/year. We observe that the changes in magnitude of TOT_E_ are generally less uncertain than those in precipitation.

The decrease in AE_P_ indicates an increase in the seasonal variability of precipitation in the regimes L_P_L_AE_, M_P_L_AE_, and H_P_L_AE_ for all RCP scenarios as detailed in Fig. 2b. Although a negligible trend in RCP 2.6 is observed in case of regimes H_P_M_AE_, H_P_H_AE_, and M_P_M_AE_, a significant increase in variability was observed in the other scenarios for these three regimes. However, although a negligible positive trend is observed in regimes L_P_M_AE_ and L_P_H_AE_ for RCP 2.6, the other scenarios exhibit a decrease in variability. Regime M_P_H_AE_, which is characterized by moderate and consistent water supply exhibits a negligible change in all scenarios. Further, the changes in AE_E_ are not as substantial as those in AE_P_. No significant trends are observed in AE_E_ for regimes H_P_H_AE_, H_P_M_AE_, H_P_L_AE_, and M_P_L_AE_. In addition, decrease in variability of evaporation is evident in regime L_P_H_AE_, L_P_M_AE_, M_P_H_AE_, and M_P_M_AE_. The higher emission scenarios display greater change in magnitude for AE_E_. Unlike with precipitation variability, there is a pattern of decreased variability exhibited by AE_E_. These contrasting future projection changes in precipitation and evaporation may result in spatially variable monthly WA (i.e., P–E)^6,31,34^. To further assess the robustness of our results, we also computed the grid-wise trends as shown in Supplementary Figure 1 for RCP 8.5 scenario. Based on this spatially explicit analysis, we see that precipitation variability is increasing over a substantially greater region (~35.6% of the land surface) than it is decreasing (~4% of the land surface). In addition, the spatial analysis indicates that this increase in variability in more prominent in regions with high variability. Evaporation variability, conversely, is decreasing over a substantially greater region (~36% of the land surface) than it is increasing over (~6% of the land surface). Therefore, both these analyses suggested an overall trend toward increasing precipitation variability along with decreasing evaporation variability, reinforcing our main conclusions. As these metrics aggregate the monthly distributions of precipitation and evaporation at the annual scale obscuring seasonal behavior, it is worthwhile to additionally investigate, as described below, how these changes are reflected in the monthly distribution of available water (determined as the difference between precipitation and evaporation—See “Methods”).

### Precipitation and evaporation role in available water change

**Fig. 3:**
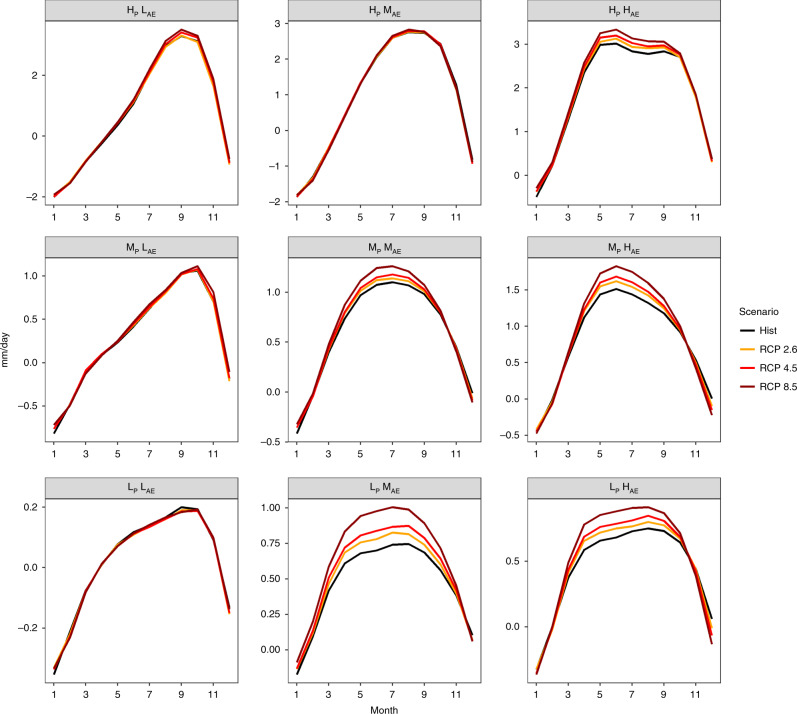
Future monthly water availability. **a** BMA ensemble monthly projected (RCP 2.6, RCP 4.5, and RCP 8.5) of available water (2070–2099) and historical (1971–2000) scenarios. Where, H, M, and L abbreviate high moderate and low values, respectively. Whereas, apportionment entropy is abbreviated as AE and annual precipitation as P.

We determine which seasonal components have contributed to altering the monthly distribution of WA (precipitation–evaporation) for each of these scenarios (See “Methods”). The BMA-weighted historical (1971–2000) monthly WA distribution and future projections (2070–2099) for each regime are shown in Fig. 3 for each of the scenarios. The projected future monthly distribution of WA for the wet seasons (8th–10th month) demonstrates that the wet season becomes wetter, a pattern that is increasingly evident in regimes L_P_H_AE_, M_P_H_AE_, and H_P_H_AE_ with a consistent water supply albeit less evident in regimes L_P_L_AE_ and M_P_L_AE_ with an inconsistent water supply (regime H_P_L_AE_ exhibits a minor increase in available water). So, while seasonally variable regimes becoming more variable in terms of precipitation, that is not the case for WA, perhaps owing to competing effects between evaporation and precipitation. The low precipitation of regime L_P_L_AE_ exhibits the least noticeable change in monthly available water, unlike the high precipitation of regime H_P_L_AE_, wherein a more noticeable increase in available water is evident during the wet season. This pattern is also sensitive to the change in radiative forcing, with greater radiative forcing corresponding to greater changes. Therefore, in regions where water supply is constrained by low-precipitation amounts and a more uneven distribution (low AE), changes in precipitation and evaporation do not affect the available water as they are more water-limited in nature. In regions with a more even distribution, however, there is a greater effect. Overall, these results indicate that the changes in monthly WA distribution are dependent on both the particular regime and the radiative forcing.

**Fig. 4:**
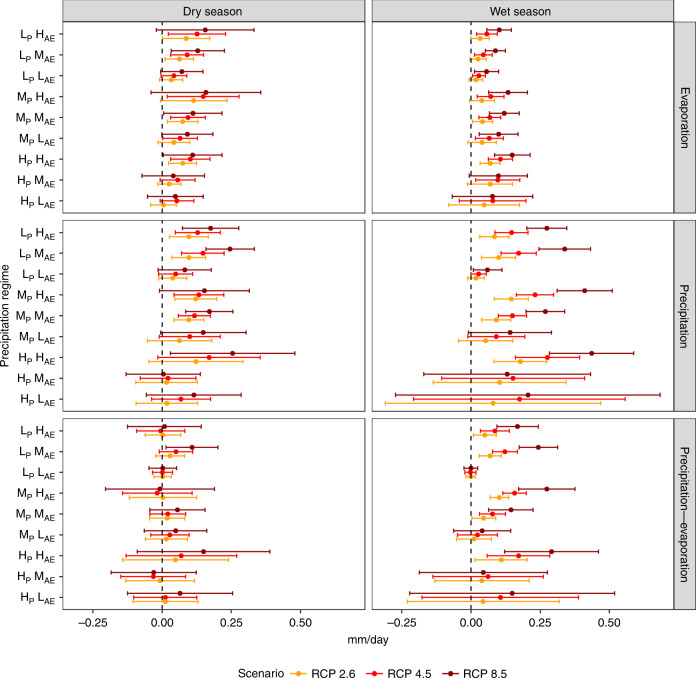
Projected seasonal changes in precipitation, evaporation, and available water. Relative changes in future precipitation and evaporation during the wet (three-month average with maximum precipitation in a year) and dry seasons (three-month average with minimum precipitation in a year) derived based on the CMIP5 scenario relative to the historical period (1971–2000). The error bars surrounding the point estimates represent the 95% confidence interval of the CMIP5 multimodel ensemble. H, M, and L abbreviate high moderate and low values, respectively. Whereas, apportionment entropy is abbreviated as AE and annual precipitation as P.

The corresponding changes in precipitation and evaporation (relative to the historical period) during the wet and dry seasons for three future emission scenarios (RCP 2.6, 4.5, 8.5) are presented in Fig. 4, illustrating the competition between water and energy balance at the seasonal scale. The role played by both wet and dry seasons in changing WA are examined in Fig. 4. In regions with high AE values (i.e., L_P_H_AE_, M_P_H_AE_, and H_P_H_AE_), higher increases in evaporation can be observed in comparison with the moderate and low AE regions, especially in the RCP 8.5 scenario. These changes are significant in the wet season but not the dry season. As noted earlier, higher emission scenarios show more pronounced evaporation increases. In the case of precipitation, regimes with high AE values in both dry and wet seasons display a pattern of increase similar to that in evaporation. But these changes are again only statistically significant in the wet season. Although greater changes in precipitation are evident in the dry season, the greater spread among models in that case hinders any confident conclusions.

The increasing wet and dry season precipitation and evaporation provide an explanation for the changes in available water as defined by the difference between precipitation and evaporation (precipitation–evaporation). In regimes where the relative magnitude of the increase in evaporation is less than that in precipitation, a significant increase in WA is observed. Such is the case with high AE regions in wet season. This increase can thus be attributed to a larger increase in precipitation than in evaporation. In addition, regions with higher AE exhibit a greater increase in WA, which imply the potential for increased flood risk in regions such as Western Europe^35^, North America^36^, and South Eastern Asia that show less-seasonal variation^16^. In the case of the dry season, an increase in WA is found in RCP 8.5 scenarios for regimes L_P_M_AE_, M_P_M_AE_, and H_P_H_AE_ but it is not statistically significant. The results in these cases are inconclusive owing to the large spread among models. This finding further highlights the interdependent role of evaporation and precipitation in changing seasonal WA. Previous studies based on spatially explicit analyses have shown that the dry seasons are becoming drier in some locations around the globe^9^. Our spatial analysis in case of RCP 8.5 scenario also indicates a decrease in P–E in Europe and northern part of North America (Supplementary Fig. 2) in agreement with Kumar et al.^10,37^.

## Discussion

Coupled changes in seasonal variation (as measured by AE) and annual mean precipitation and evaporation significantly impact the spatio-temporal distribution of WA. By using appropriate thresholds applied to seasonal variations and annual means of historical precipitation data, nine land regions were identified that are characterized by different water supply regimes.

Spatially aggregated future trends over these regimes as applied to the CMIP5 future projections indicate an increase in precipitation annual means in all land regimes across the globe, with higher increases in RCP 8.5 as reported in previous studies^6–8,38^. Most strikingly, annual mean evaporation is found to be increasing in all regimes, indicating a pattern of intensified response to increased water demand across all regions. Furthermore, an increase in seasonal precipitation variation is observed, especially in regions that already exhibit greater seasonal variation in precipitation. This indicates a pattern of seasonally variable regimes becoming more variable regarding precipitation. This pattern is consistent with previously reported observations of the wet season becoming more wet and the dry season becoming more dry^39,40^. In the case of wet seasons becoming wet, vertical moisture advection and evaporation play a key role^40,41^. Therefore, we anticipate that the observed pattern may be owing to regional changes in moisture advection and evaporation. However, we do not observe such a pattern in the seasonal variability of evaporation. We found a decrease in seasonal evaporation variation in various regimes of the global land area. This would imply that regions that already have inconsistent water supply owing to high seasonal variation in precipitation might experience even more inconsistent WA.

Similar to what is observed with precipitation, an increase in the annual mean evaporation (TOT_E_) is observed in all regimes with the smallest increase observed in regime L_P_L_AE_, as found in previous studies^41^. Increases in annual mean evaporation over the land surface can be attributed to the increase in temperature in the CMIP5 future projections^42^ and also to increase in precipitation. The seasonal changes in evaporation, however, are tied to corresponding seasonal changes in surface relative humidity contrast^42,43^. Also, the increase in TOT_E_ is smaller than in TOT_P_, a finding that is consistent with previous studies^31,42^. We also examined the projected monthly distribution of WA as measured by precipitation minus evaporation (P–E) across the classified nine precipitation regimes. Our results highlight a clear signal of increased WA in the wet season especially in regimes of less-seasonal variation in precipitation.

We assessed the role of precipitation and evaporation characteristics in the changing monthly distributions of WA. Our results indicate that the increases in WA in wet seasons is controlled by changes in both precipitation and evaporation. The combination of changes in precipitation and evaporation might result in an overall increase in WA, which is more pronounced during the wet season and is expected to yield spatially variable annual WA consistent with previous studies^37,44,45^. However, we show that these changes may also be dependent upon the specific regime as determined by both seasonal variation and mean precipitation changes. Even though our results indicate changes in long-term WA, it is unclear what implications our findings might hold for hydrologic extremes, owing to limitations in the ability of current generation coupled climate models to capture the key drivers of persistent weather extremes^46,47^.

Overall, these changes in precipitation characteristics impact not only annual WA but also its spatio-temporal distribution. Concomitant changes in the mean and seasonal variation in precipitation may imply significant and varied shifts in phenology^48,49^, reservoir management^3^, and ecosystem function depending upon the water supply regimes. The framework provided by our study complements traditional approaches used to study seasonal variability in different hydroclimatic regimes^50,51^. Future efforts will use this framework to quantitatively assess the implications of projected changes in seasonal and annual mean precipitation on streamflow regimes, providing further relevance to issues involving societal water use and demand.

### Data

Observed precipitation gridded data from GPCC^52^ at a resolution of 2.5 × 2.5^◦^ for the period of 1901–2005 at monthly scale is used. Conversely, the observed terrestrial evaporation measurements were obtained from Global Land Evaporation Amsterdam Model (GLEAM) data set available for the period of 1980–2015^53^ at a daily scale. This data set incorporates the Priestley and Taylor equation to calculate the potential evaporation based on observations of surface net radiation and near surface air temperature. A multiplicative evaporative stress factor estimated from satellite estimates is then used to convert the calculated potential evaporation values to actual evaporation. A more-detailed calculation procedure is available in the work of Martens et al.^53^.

The future projected data from 21 different general climatic models under the CMIP5 version for three different RCP scenarios are used. Each of these scenarios are distinguished by their radiative forcing increases by the end of the current century (RCP 2.6, 4.5, and 8.5) relative to pre-industrial values (Supplementary Table 1). These scenarios correspond roughly to a 2 °C stabilization, and 3.5 °C and 5 °C global temperature increase, respectively, by the end of the century. These CMIP5 models are selected based on the common availability of both precipitation and evaporation variables for historical and projected scenarios.

## Methods

### Annual precipitation and evaporation characteristics

After acquisition of the data, both the observed and modeled data are interpolated to a common 2.5 × 2.5° grid from their respective original grids. We assessed the seasonal variation of monthly precipitation and evaporation using an information theory metric called as AE. Unlike the parametric coefficient of both variation and standard deviation, this metric is non-parametric and may even encompass high-order moments^3^. Moreover, information theory metrics have been widely used as a measure of rainfall seasonality in both hydrologic and climatological contexts^3,24,26,27^. Therefore, to estimate AE for either precipitation or evaporation over a year *k*, the aggregated annual quantities during the 12 months indexed are computed by summing the monthly values (*x*) over all the months in a year as

1 $$X = \mathop {\sum}\limits_{i = 1}^{12} {x_i}$$

where *X* is the aggregated value of either the precipitation or evaporation. Subsequently, the AE is calculated as

2 $$AE = - \mathop {\sum}\limits_{i = 1}^{12} {\left( {x_i/X} \right)\log _2\left( {x_i/X} \right)}$$

By definition, both Eqs. (1) and (2) state that when either the amount of annual precipitation or evaporation is quite evenly apportioned to each of the 12 months with the probability of 1/12, Eq. (2) assumes the maximum value of log_2_12. Conversely, the minimum value of AE = 0 occurs when the apportionment is extended to only 1 month of the 12 month cycle with a probability of 1, thus indicating the assumption of a new value by AE within a finite range of 0 and log_2_12. Therefore, in a year, the less variable the monthly precipitation or evaporation, the higher the AE value. Using this definition, the seasonal variability of precipitation (AE_P_) and evaporation (AE_E_) is estimated for the study period. The aggregation of both the precipitation (TOT_P_) and evaporation (TOT_E_) is then used to calculate the annual precipitation and evaporation during these 12 months.

### Definition of global classification of precipitation regimes

The existing atmospheric climate classifications are based on broad precipitation, temperature, evapotranspiration, and biosphere characteristics^21–23^. However, even though the seasonal variability significantly changes across the global land regions^26,27^, it is often not considered for classification from a non-parametric standpoint. For instance, the occurrence of substantial precipitation with an even monthly distribution will indicate a consistent and adequate supply of atmospheric water throughout the year. Similarly, an occurrence of substantial precipitation with an uneven monthly distribution will indicate surplus water supply during a particular period of a given year and deficit water supply during another period in a given year. Therefore, a failure to consider the seasonal variability of precipitation magnitude may well lead to a misrepresentation of the actual water supply conditions. Therefore, in this study, in addition to the precipitation magnitude, the seasonal variability was also included for purposes of classifying the global land regions into distinct water supply regimes. Here, the global land was divided into nine regimes of varying magnitudes of observed annual precipitation and seasonal variability based on the period between the years 1971 and 2000. These regimes are derived based on a combination of seasonal variabilities (i.e., AE) and magnitude using the threshold concept: with high (>Pr_70_), moderate (between Pr_30_ and Pr_70_) and low (<Pr_30_), in which Pr_i_ represents the ith percentile of either the annual magnitude or the seasonal variability. The use of the 30th and 70th percentiles is based upon the wet and dry region definitions adopted by Allan et al.^54^ and subsequently implemented in Liu and Allan^55^. A similar threshold based classification of various dry and wet global regions has been used elsewhere with success^56–58^. In using this approach, we have coupled the characteristics of both annual magnitude and seasonal variability into distinct regimes based on global percentile thresholds. As a result, this definition can capture the precipitation characteristics that might have been previously omitted in those definitions based on absolute thresholds, separate precipitation magnitude and seasonal variability considerations and regional definitions^59^. The classification of these regimes also do not explicitly consider the natural landscape (e.g., rainforest, deserts) or the human-experienced climate events (e.g., monsoons) as suggested by Trewartha^54^. As we base our analysis on both precipitation and evaporation, this scheme provides a necessary and rather simple configuration for the relative assessment of spatially aggregated changes in both precipitation and evaporation characteristics for this study. All the derived precipitation and evaporation characteristics for CMIP5 as well as the observed data over these regimes are spatially aggregated.

### Bayesian model averaging

As the CMIP5 models exhibit varying levels of accuracy regarding simulating historical hydrologic cycles, the BMA methodology^52^ is utilized to assign higher weights to better performing models. For this purpose, we computed the yearly precipitation and evaporation totals and AE, from the CMIP5 models and observed data sets, i.e., precipitation from GPCC and evaporation dataset from GLEAM over each precipitation regime. Then the BMA approach is utilized to determine the optimal weights for each CMIP5 model on their ability to replicate the spatial patterns of TOT_P_, TOT_E_, AE_P_, and AE_E_. The BMA approach calculates ensemble of the considered CMIP5 models by assigning weights based on the performance of models in comparison with the observations^52,60^. In this approach, the probability density function (*g*) of our interest variable, $Y \in \left\{ {P,E,AE_P,AE_E} \right\}$ from the observed data, which is conditioned upon the 21 CMIP5 simulations is expressed as:

3 $$g\left( {Y|Y_1,...,Y_{21}} \right) = \mathop {\sum}\limits_{i = 1}^{21} {w_if\left( {Y|Y_i} \right)}$$

where *w*_*i*_ is the optimal weight for the *i*th CMIP5 model, *f*(*Y*|*Y*_*i*_) is a PDF of the gamma distribution of *Y*_*i*_. The general selection of the BMA mixture probability distribution is normal with the gamma distributions based on the suggestion of Vrugt et al.^60^ and Raftery et al.^52^. Therefore, in this study, a separate analysis is undertaken for both of the prior distributions. The BMA ensemble weighted mean and standard deviation is thus expressed as:

4 $$En = \mathop {\sum}\limits_{i = 1}^{21} {w_i * Y_i}$$

5 $$RMSD = \left[ {\mathop {\sum}\limits_{i = 1}^{21} {w_i\left( {Y_i - EN} \right)} } \right]^{1/2}$$

### Estimation of BMA weights

The optimal weights w_*i*_, *i* = 1, …, 21 are estimated with a maximum log-likelihood function, which is expressed as:

6 $$l\left( {w_1, \ldots ,w_{21}} \right) = \mathop {\sum}\limits_{j = 1}^N {\log \left( {\mathop {\sum}\limits_{i = 1}^{21} {w_ig\left( {Y_j\left| {Y_{i,j}} \right.} \right)} } \right)}$$

where *j* = {1, … *N*} are observations of the considered variable. Regarding precipitation, we use the mean annual estimates from 1971 to 2000 for estimating the optimal weights. Regarding evaporation, we only use the annual characteristics from 1980 to 2005. A Markov Chain Monte Carlo algorithm^60^, which maximizes the log-likelihood function is used for estimating the weights. This algorithm in turn generates *N* different Markov Chains, placed separately as rows with each chain represented as a 22-dimensional vector *θ* = {*w*_*1*_*, w*_*2*_*,…, w*_*21*_*, σ*^*2*^}. The candidate maximum likelihood point is then sampled from a prescribed distribution, depending on the precipitation regime. Next, in accordance with the Metropolis acceptance probability, the candidate maximum likelihood point is either accepted or rejected. If accepted, the chain moves to the next candidate model; otherwise the chain repeats the process until the optimal weight for the candidate model is estimated. Clearly, the BMA weights obtained through maximum likelihood using this procedure is comparatively more accurate than the weights obtained using the Expectation–Maximization (EM) algorithm recommended by Raftery et al.^52^. A thorough description of this algorithm is presented in Vrugt et al.^60^. The estimated BMA weights are listed in Supplementary Table 2.

### Performance and evaluation of BMA multimodel ensembles over precipitation regimes

The performance of the BMA multimodel ensemble was assessed in terms in replicating the spatial pattern across the land regions. After obtaining the optimal weights (shown in Supplementary Table 2), we computed the multimodel ensemble using Eq. (4) and evaluated the performance by the metrics of the Pearson correlation. The results indicate that in terms of the TOT_P_, TOT_E_, an *R*^2^ values of 0.94 and 0.92, respectively, is obtained. Whereas in case of AE_P_ and AE_E_ an *R*^2^ of 0.91 and 0.85 are obtained. These values further indicate that ensemble mean of the 21 GCMs, which are studied could adequately replicate the spatial patterns.

### Assessment of projected changes

The projected changes of TOT_P_, TOT_E_, AE_P_, and AE_E_ were determined using the non-parametric Theil-Sen estimator^61^. We first estimated the linear trends using Theil-Sen estimator ($\Delta Y$) and the BMA ensemble trend with an uncertainty is expressed as

7 $$\Delta Y_{BMA} = \mathop {\sum}\limits_{i = 1}^{21} {w_i * \Delta Y_i}$$

8 $$\Delta Y_{RMSD} = \left[ {\mathop {\sum}\limits_{i = 1}^{21} {w_i\left( {\Delta Y_i - \Delta Y_{BMA}} \right)} } \right]^{1/2}$$

thus, permitting the estimates of the upper and lower confidence limits as:

9 $$\Delta Y_{UC} = \Delta Y_{BMA} + 2 * \Delta Y_{RMSD}$$

10 $$\Delta Y_{LC} = \Delta Y_{BMA} - 2 * \Delta Y_{RMSD}$$

If the changes are distributed as a Gaussian density function, then these bounds imply a ~95% confidence interval.

### Available water

The difference between monthly precipitation and evaporation variables is deemed reliable for approximating the potential available water for human and ecological consumption. Here, we estimate the average net available water in 1 month for the historical period of 1971–2000 and for the future scenario of 2070–2099, which is expressed as:

11 $$WA_{hist}^m = \frac{{\mathop {\sum}\nolimits_{y = 1970}^{2000} {\left( {r_{m,y} - e_{m,y}} \right)} }}{{30}}$$

12 $$WA_{rcp}^m = \frac{{\mathop {\sum}\nolimits_{y = 2070}^{2100} {\left( {r_{m,y} - e_{m,y}} \right)} }}{{30}}$$

where *r*_*m,y*_ and *e*_*m,y*_ represent the precipitation and evaporation in month *m* and year *y*. We then compare the monthly net available water of historical and future scenarios (RCP 2.6, 4.5, and 8.5) from each GCM model through a spatial averaging over respective regimes. The denominator 30 represents the total number of years taken into consideration. In case of WA, we used the averaged BMA weights for each model and estimated the monthly distribution of WA.

To explore the role of seasonal components in terms of altering the monthly climatology of available water seasonal variability within a hydrologic year, we first extracted wet and dry precipitation and evaporation components. We assumed a 3-month period in which the maximum (minimum) of available water occurs as wet (dry) seasons^40^ using the same base period of 1971–2000. We estimated the changes in both the wet and dry seasons as the difference between the future scenarios (2070–2099) and the mean historical (1971–2000) spatially aggregation over each regime. The BMA weights were applied and the uncertainty was calculated using Eqs. (7–10).

## Supplementary information

Supplementary Information

## Data Availability

The data sets analyzed during the current study are available at Earth System Grid Federation (ESGF) Peer-to-Peer (P2P) enterprise system [https://esgf-node.llnl.gov/projects/esgf-llnl/]. The Observed monthly GPCC precipitation is available at https://opendata.dwd.de/climate_environment/GPCC/html/fulldata-monthly_v2018_doi_download.html. The evaporation data set is available at https://www.gleam.eu/.

## Code availability

The accompanying MATLAB source code for Bayesian model averaging utilized in this study is available at (http://faculty.sites.uci.edu/jasper/software).
