# Supplementary material for: Climate change will affect global water availability through compounding changes in seasonal precipitation and evaporation
Source: Nat Commun. 2020 Jun 23;11:3044. doi: 10.1038/s41467-020-16757-w (PMC7311549; doi:10.1038/s41467-020-16757-w)
Supplement: Supplementary file 1 — Supplementary Information [file 41467_2020_16757_MOESM1_ESM.pdf]

Supporting Information

Climate Change Will Affect Global Water Availability through Joint Changes in Seasonal Precipitation and Evaporation

Konapala et al.

Supplementary Table 1: GCM models used for analysis (Only r1i1p1 realizations are selected among these models)

GCM Model	Hist	RCP 8.5	RCP 4.5	RCP 2.6
bcc-csm1-1	✓	✓	✓	✓
bcc-csm1-1-m	✓	✓	✓	✓
BNU-ESM	✓	✓	✓	✓
CanESM2	✓	✓	✓	✓
CCSM4	✓	✓	✓	✓
CESM1-CAM5	✓	✓	✓	✓
CNRM-CM5	✓	✓	✓	✓
CSIRO-Mk3-6-0	✓	✓	✓	✓
FIO-ESM	✓	✓	✓	✓
GISS-E2-H	✓	✓	✓	✓
HADGEM2-ES	✓	✓	✓	✓
IPSL-CM5A-LR	✓	✓	✓	✓
IPSL-CM5A-MR	✓	✓	✓	✓
MIROC5	✓	✓	✓	✓
MIROC-ESM	✓	✓	✓	✓
MIROC-ESM-CHEM	✓	✓	✓	✓
MPI-ESM-LR	✓	✓	✓	✓
MPI-ESM-MR	✓	✓	✓	✓
MRI-ESM1	✓	✓	✓	✓
NorESM1-M	✓	✓	✓	✓
NorESM1-ME	✓	✓	✓	✓

Supplementary Table 2: BMA weights of GCM models

**GCM Model**	**TOT_P**	**TOT_E**	**AE_P**	**AE_E**
bcc-csm1-1	0.035	0.042	0.043	0.047
bcc-csm1-1-m	0.052	0.040	0.050	0.050
BNU-ESM	0.052	0.051	0.060	0.050
CanESM2	0.059	0.069	0.056	0.049
CCSM4	0.036	0.039	0.041	0.049
CESM1-CAM5	0.046	0.043	0.054	0.049
CNRM-CM5	0.042	0.045	0.041	0.048
CSIRO-Mk3-6-0	0.018	0.022	0.039	0.042
FIO-ESM	0.081	0.066	0.055	0.047
GISS-E2-H	0.044	0.064	0.036	0.043
HADGEM2-ES	0.044	0.064	0.036	0.042
IPSL-CM5A-LR	0.044	0.030	0.044	0.049
IPSL-CM5A-MR	0.043	0.030	0.043	0.049
MIROC5	0.041	0.037	0.052	0.049
MIROC-ESM	0.060	0.057	0.060	0.048
MIROC-ESM-CHEM	0.061	0.057	0.059	0.048
MPI-ESM-LR	0.051	0.065	0.052	0.053
MPI-ESM-MR	0.050	0.035	0.047	0.048
MRI-ESM1	0.048	0.035	0.047	0.049
NorESM1-M	0.050	0.059	0.043	0.048
NorESM1-ME	0.046	0.050	0.043	0.046

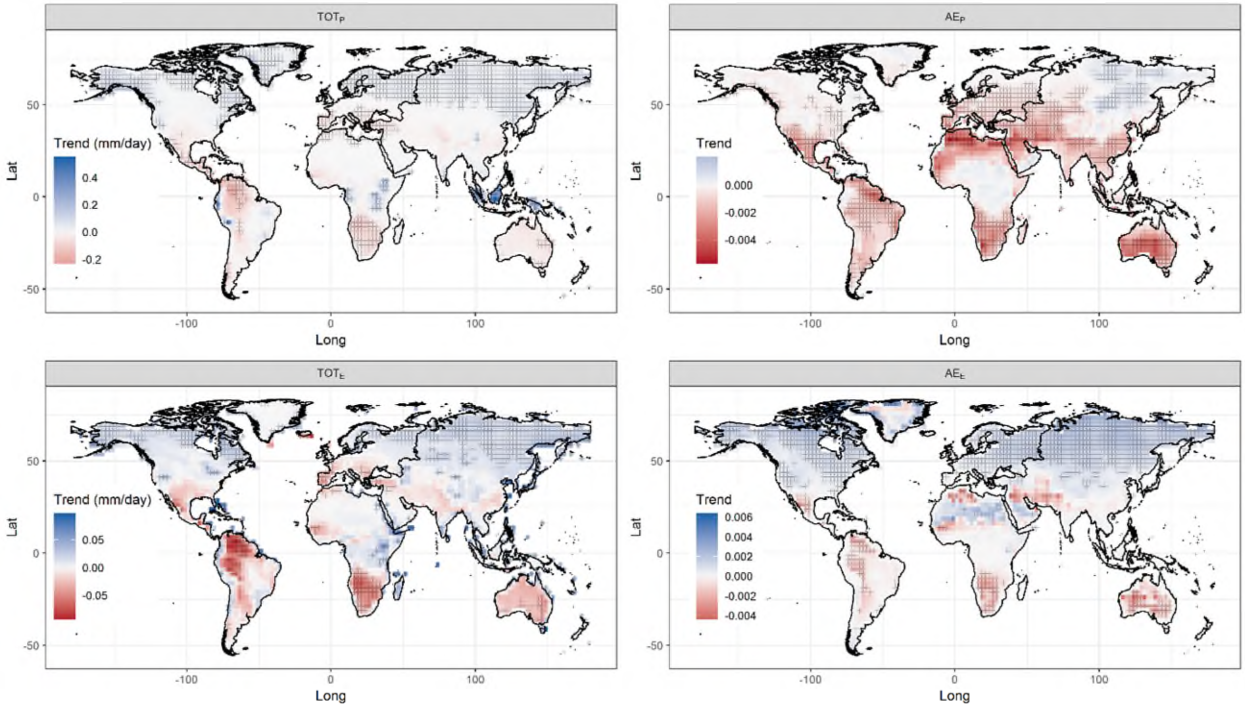

Supplementary Figure 1: **Trends in precipitation and evaporation characteristics**: Spatial Maps of trends in total precipitation (TOTP), AE of precipitation (AEP), total evaporation (TOTE), and AE of evaporation (AEE) in case of RCP 8.5 scenario (2005-2100). Linear trends are estimated using Theil-Sen estimator as described in the main text. The stippling indicates statistically significant (p value <0.05) grids.

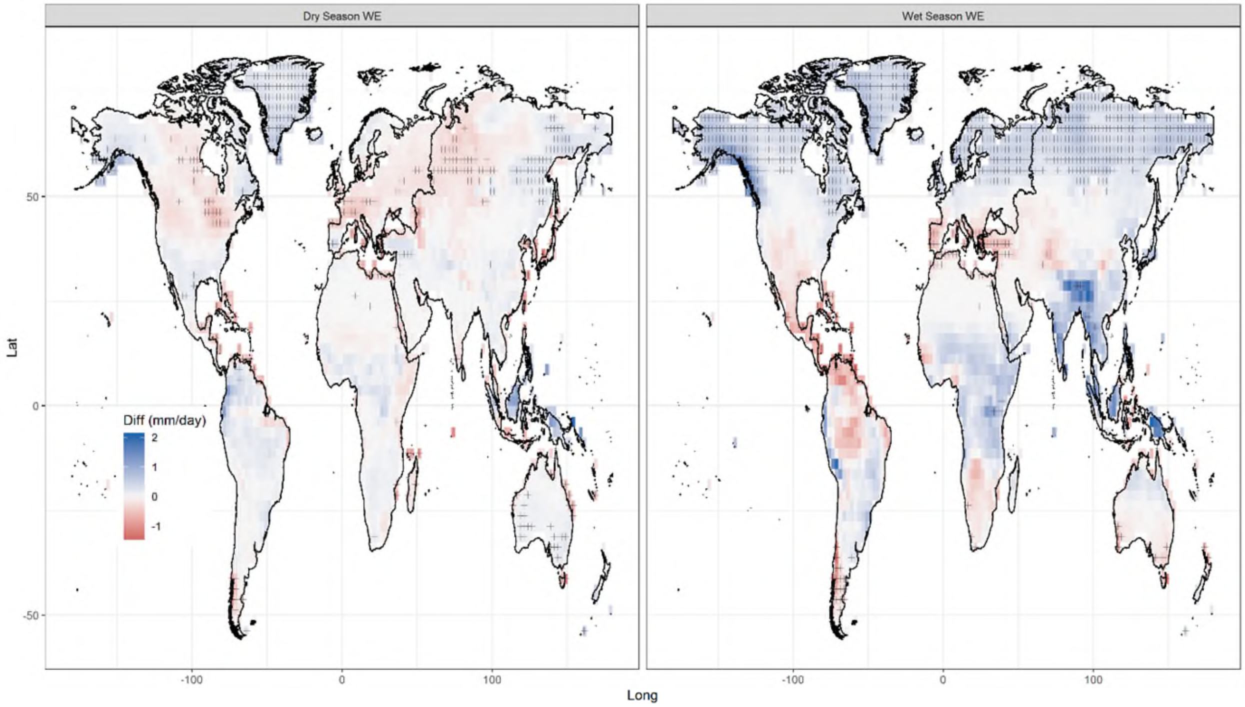

Supplementary Figure 2: **Changes in future projections of available water**: Spatial Maps of wet season and dry season water availability (WA) based on the difference between RCP 8.5 scenario (2070-2100) and historical scenario (1970-2000). The stippling indicates statistically significant (p value <0.05) grids.
